# An integrated model for prognosis in vulvar squamous cell carcinoma

**DOI:** 10.1186/s12885-023-11039-2

**Published:** 2023-06-12

**Authors:** Tao Zhang, Yingfan Zhu, Jie Luo, Juanqing Li, Shuang Niu, Hao Chen, Feng Zhou

**Affiliations:** 1 Department of Gynecology, Zhejiang University School of Medicine Women’s Hospital, Hangzhou, 310006 China; 2 Department of Pathology, University of Texas Southwestern Medical Center, Dallas, TX 75390 USA; 3 Department of Pathology, Zhejiang University School of Medicine Women’s Hospital, Hangzhou, 310006 China; 4 Present Address: Department of Pathology, International Peace Maternity and Child Health Hospital Affiliated to Shanghai Jiao Tong University School of Medicine, Shanghai, 200030 China

**Keywords:** Vulvar squamous cell carcinoma, Prognosis, Nomogram, HPV, Ki-67, CD8, PD-L1, p53

## Abstract

**Background:**

Vulvar squamous cell carcinoma (VSCC) is a relatively rare gynecologic cancer. Unlike cervical squamous cell carcinoma (CSCC), in which nearly all cases are caused by HPV infection, most VSCCs are HPV-independent. Patients with VSCC also have worse overall survival (OS) than those with CSCC. Unlike CSCC, the risk factors of VSCC have not been extensively studied. Here, we investigated the prognostic values of clinicopathological parameters as well as biomarkers in patients with VSCC.

**Methods:**

In total, 69 cases of VSCC accessions were selected for analysis between April 2010 and October 2020. The risk factors of VSCC were screened using Cox models to establish nomograms for predicting survival outcomes.

**Results:**

Following the multivariate COX model for OS, independent predictors including advanced age (hazard ratio [HR] 5.899, p = 0.009), HPV positivity (HR 0.092, p = 0.016), high Ki-67 index (HR 7.899, p = 0.006), PD-L1-positivity (HR 4.736, p = 0.077), and CD8 + tumor-infiltrating lymphocytes (TILs) (HR 0.214, p = 0.024) were included in the nomogram for OS; multivariate COX model for progression-free survival (PFS) was used to screen prognostic factors including advanced age (HR 2.902, p = 0.058), lymph node metastasis (HR 5.038, p = 0.056), HPV positivity (HR 0.116, p = 0.011), high Ki-67 index (HR 3.680, p = 0.042), PD-L1-positivity (HR 5.311, p = 0.045), and CD8 + TILs (HR 0.236, p = 0.014) to establish the PFS nomogram model. Based on the C-index (0.754 for OS and 0.754 for PFS) from our VSCC cohort and the corrected C-index (0.699 for OS and 0.683 for PFS) from an internal validation cohort, the nomograms demonstrated good predictive and discriminative ability. Kaplan-Meier curves also supported the excellent performance of the nomograms.

**Conclusion:**

Our prognostic nomograms suggested that (1) shorter OS and PFS were associated with PD-L1-positivity, high Ki-67 index, and low CD8 + TILs; (2) HPV-independent tumors were associated with poorer survival outcome, and mutant p53 status showed no prognostic significance.

**Supplementary Information:**

The online version contains supplementary material available at 10.1186/s12885-023-11039-2.

## Introduction

Vulvar cancer is rare, constituting 5–8% of gynecological malignancy [1]. According to the statistics of the GLOBOCAN, 45,240 new cases and 17,427 deaths from vulvar cancer were reported globally in 2020 [2]. More than 90% of vulvar cancer is squamous cell carcinoma (SCC) and usually affects post-menopausal women aged 68 or older [1, 3]. Vulvar SCC (VSCC) is classified into two types (HPV-associated and HPV-independent) based on the etiologies and their two distinct precancerous lesions, HPV-associated usual vulvar intraepithelial neoplasia (uVIN) / vulvar high-grade squamous intraepithelial lesion (HSIL) and HPV-independent differentiated vulvar intraepithelial neoplasia (dVIN) respectively [1, 4]. uVIN / HSIL is associated with infection of high-risk human papillomavirus (hrHPV) and commonly seen in younger women [3, 4], while dVIN is HPV-independent mostly arising from lichen sclerosus (LS) in older woman [3, 4]. HPV-associated VSCC shares many risk factors as cervical SCC, such as low socioeconomic status, smoking history, other sexually transmitted diseases, immunodeficiency, etc. [3, 5]. On the other hand, dVIN is the precancer of HPV-independent VSCC and has a higher risk of developing into SCC than uVIN / HSIL [6]. Furthermore, TP53 mutant status can be used to further stratify HPV-independent VSCC into two prognostic subtypes (p53 mutant and p53 wild-type) [7]. Cross-pathogenetic pathways are also possible for these two VSCC types [8].

In 2021, the International Federation of Gynecology and Obstetrics (FIGO) published a new revision to the 2009 FIGO staging of vulvar cancer based on the analysis of prospectively collected survival data on carcinoma of the vulva [9]. The 2021 vulvar cancer staging redefines depth of invasion, uses the same definition for lymph node metastases as cervical cancer, and includes cross-sectional imaging findings similar to cervical cancer [9]. However, the latest staging revision alone may not be enough, and other clinical parameters should be considered for the clinical management of vulvar cancer. For instance, due to unresectable or obvious margin positive, postoperative radiotherapy may be required depending on the distance between the tumor and vulvar midline structures (clitoris, anus, or urethra), which were not included in the current staging scheme [1].

Currently, anti-PD-1 and anti-PD-L1 inhibitors have been successfully used in the treatment of gynecological cancers including cervical and endometrial cancers [10–13]. However, by far, only limited studies investigated the expression of PD-L1 and its prognostic value in vulvar cancer [14–17]. Furthermore, the relationship among the expression of PD-L1, clinicopathological parameters, and survival of vulvar cancer remains unclear. This study aims to establish an integrated model comprising clinicopathological parameters, HPV status, and PD-L1 expression to predicate the clinical outcomes of VSCC, in hope that better risk stratification of patients could direct potentially less aggressive treatments in low-risk patients to decrease morbidity and improve quality of life.

## Materials and methods

### Patient samples and database

We conducted a retrospective analysis of 69 patients with VSCC between April 2010 and October 2020 at the Women’s Hospital, School of Medicine, Zhejiang University. The inclusion criteria were as follows: (1) staging surgery as the initial therapy (modified radical vulvectomy + sentinel lymph node biopsy/bilateral inguinofemoral lymphadenectomy); (2) VSCC proved by pathological diagnosis; and (3) with complete follow-up information. The exclusion criteria were: (1) combined with other tumors; (2) unknown status of lymph node metastasis (LNM); and (3) unknown HPV status. Two senior pathology physicians reviewed all the cases independently and the stage of all cases were revised according to the 2021 FIGO staging revision [1, 9].

### Immunohistochemistry (IHC)

IHC was performed according to the previous studies [18–20]. Sections were cut at 4 µm from each paraffin block and were dried in an oven for 1 hour at 56 to 60°C. Deparaffinization, rehydration, and target retrieval were performed according to the manufacturer by fully submerging the slides in preheated (65°C) EnVision FLEX Target Retrieval Solution for antibodies, and incubating slides at 97°C for 20 minutes. Then the slides were immediately submerged in a wash buffer for 5 minutes at room temperature. With antigen retrieval, the slides were placed in the Autostainer Link 48 platform (Dako/Agilent, and subjected to FLEX peroxidase block for 5 minutes. Then, slides were incubated with primary antibodies: p16 (GM501, GeneTech, Shanghai, China), p53 (DO-7, GeneTech), Ki-67 (GM001, GeneTech), PD-L1 (22C3, Dako, Santa Clara, CA) and CD8 (C8/144B, Dako, Santa Clara, CA). Incubation time was according to primary antibody (20 to 60 minutes). After incubation with the primary antibody, incubation with the EnVision™ FLEX + Mouse LINKER anti-mouse linker antibody was performed for 30 minutes. Subsequently, all slides were rinsed in wash buffer for 5 minutes and then incubated with the EnVision™ FLEX HRP visualization reagent for 30 minutes at room temperature. After rinsing in wash buffer for 5 minutes, the enzymatic conversion of the subsequently added 3,3’-diaminobenzidine (DAB) tetrahydrochloride chromogen was performed for 10 min at room temperature followed by DAB enhancer for 5 min at room temperature.

### HPV status and IHC scoring

P16 expression was graded as previously published [21]: (1) negative (score 0) when diffusely weak or absent; (2) patchy positive (score 1) when nuclear and cytoplasmic staining detected in less than 50% of tumor cells; (3) overexpressed (score 2) when diffuse nuclear positivity of tumor cells present. Tumors with scores 0 and 1 were defined as HPV-independent (p16-negative), while tumors with score 2 were defined as HPV-associated (p16-positive). Cases with discordant HPV-PCR and p16-IHC results were excluded from the study as these cases might influence the accurate subclassification of the VSCC. As previously described [17], p53 detected in > 50% of tumor nuclei or complete absence (null type) was considered as p53 mutant, while less than threshold tumor was considered as p53 wildtype. The cutoff value for Ki-67 was also 50%, where positive tumor nuclei ≤ 50% were considered Ki-67 low, and positive tumor nuclei > 50% as Ki67 high. The combined positive score (CPS) is used for the evaluation of PD-L1 expression of solid tumors [10]. CPS was calculated as the number of PD-L1-staining cells (tumor cells, lymphocytes, and macrophages) divided by the total number of viable tumor cells, multiplied by 100. Both scores ranged from 0 to 100. A cutoff score of ≥ 1 for CPS defined PD-L1 positivity. Open-source QuPath software (v0.3.2) was used to detect and annotate CD8 + tumor-infiltrating lymphocytes (TILs) [22]. CD8 + TILs density was calculated as total CD8 + cells divided by the area (mm^2^) comprising the tumor and areas in direct contact with the tumor periphery (within one 20x field from the tumor nest edge). Based on the median cell number (20.0 cells/mm^2^), VSCC cases were divided into CD8 + TILs low or high-density groups.

### Statistical analysis

The correlations among clinical features were analyzed by Spearman’s test. For cross-tables, two-sided Fisher’s exact or Chi-square tests were used. Cox regression was used for univariate and multivariate analysis. Nomograms were generated using multivariate Cox regression coefficients. The discrimination of nomograms was evaluated by the C-index [23] and calibration curves. Calibration curves were used to express the relationship between the actual outcome frequencies and the predicted probabilities, with a bootstrapped sample of the study group. Kaplan-Meier curves were used to stratify tumor cases using various cut-off values calculated from the nomograms and to assess the performance of models. In this study, p-value < 0.05 was considered significant. The statistical analyses were performed using software R (version 4.1.0; http://www.r-project.org). The packages of R software were used for statistical analyses and data visualization as follows: “gmodels” for Fisher’s exact or Chi-square tests; “survival” and “survminer” for Cox regression; “rms” and “nomogramEx” for nomograms, calibration plots and calculating C-index and predicted score. “ggplot2” and “ggpubr” for Kaplan-Meier plots.

## Results

### Clinicopathological characteristics, and correlation of IHC markers with clinicopathological parameters

A summary of the clinicopathological characteristics of VSCC cases and the correlation between the biomarkers and individual clinicopathological parameters were presented in Table 1. A total of 69 patients with VSCC were included in this study, and the median follow-up was 72 (range 12–156) months. The median age of VSCC patients was 67 (range 29–91) years. Among 69 patients, 53 (76.8%) cases were FIGO stage I, 45 (65.2%) cases were grade 1 tumor, 63 (91.3%) cases were lymph node metastasis (LNM) negative, and 51 (73.9%) cases were HPV-independent (negative). Among 69 tumors, 30 (43.5%) were classified as p53 mutant, 27 (39.1%) were classified as Ki-67 high, 57 (82.6%) were classified as PD-L1 positive, and 48 (69.6%) were classified as CD8 + TILs high. As cases with positive LNM were classified as advanced stage, it was not surprising that the FIGO stage of tumor and LNM positivity had a moderately positive correlation (r = 0.440, p < 0.001) (Table S1). The pairwise comparisons of other clinical variables were not statistically significant.

**Table 1:** Association of risk markers with main clinicopathological features

Variable	n (%)		P53			Ki-67			TPS			CPS			CD8		
			Wildtype	Mutant	p-value	Low	High	p-value	< 1%	≥ 1%	p-value	< 1	≥ 1	p-value	Low	High	p-value
Age	< 67 yrs	34 (50.3)	19	15	0.916	18	16	0.184	15	19	0.555	6	28	0.956	9	25	0.481
	≥ 67 yrs	35 (50.7)	20	15		24	11		13	22		6	29		12	23	
FIGO Stage	I	53 (76.8)	31	22	0.548	32	21	0.879	24	29	0.148	12	41	0.055	20	33	**0.027**
	II - III	16 (23.2)	8	8		10	6		4	12		0	16		1	15	
Grade	1	45 (65.2)	26	19	0.773	31	14	0.062	19	26	0.704	9	36	0.521	17	28	0.070
	2–3	24 (34.8)	13	11		11	13		9	15		3	21		4	20	
LMN	Negative	63 (91.3)	36	27	1.000	39	24	0.672	26	37	1.000	12	51	0.581	21	42	0.167
	Positive	6 (8.7)	3	3		3	3		2	4		0	6		0	6	
HPV status	Negative	51 (73.9)	23	28	**0.001**	39	12	**< 0.001**	19	32	0.344	9	42	1.000	13	38	0.133
	Positive	18 (26.1)	16	2		3	15		9	9		3	15		8	10	
P53	Wildtype	39 (56.5)	-	-	-	24	15	0.897	19	20	0.117	6	33	0.616	16	23	**0.029**
	Mutant	30 (43.5)	-	-		18	12		9	21		6	24		5	25	
Ki-67	Low	42 (60.9)	24	18	0.897	-	-	-	17	25	0.983	6	36	0.518	14	28	0.514
	High	27 (39.1)	15	12		-	-		11	16		6	21		7	20	
TPS	< 1%	28 (40.6)	19	9	0.117	17	11	0.983	-	-	-	6	22	0.527	12	16	0.064
	≥ 1%	41 (59.4)	20	21		25	16		-	-		6	35		9	32	
CPS	< 1	12 (17.4)	6	6	0.616	6	6	0.518	6	6	0.527	-	-	-	7	5	**0.036**
	≥ 1	57 (82.6)	33	24		36	21		22	35	-	-	-		14	43	
CD8	Low	21 (30.4)	16	5	**0.029**	14	7	0.514	12	9	0.064	7	14	**0.036**	**-**	**-**	**-**
	High	48 (69.6)	23	25		28	20		16	32		5	43		-	-	

We further investigated the relationship between clinical characteristics and the expression of markers (summarized in Table 1). The percentage of CD8 + TILs high tumors in advanced group (FIGO stage II-III) is significantly higher than that in FIGO I group (93.8%, 15/16 vs. 62.3%, 33/53, p = 0.027). The percentage of p53 mutant tumors was significantly higher in HPV-independent group than in HPV-associated group (54.9%, 28/51 vs. 11.1%, 2/18, p = 0.001). On the other hand, the percentage of Ki67 high tumors was significantly higher in HPV-associated group than in HPV-independent group (83.3%, 15/18 vs. 23.5%, 12/51, p < 0.001).

We also analyzed the relationship between the expression of IHC markers and CD8 + TILs (Table 1). CD8 + TILs appeared to be positively associated with p53 mutant status and PD-L1 positivity (p = 0.029 and p = 0.036, respectively). No statistically significant correlation was noted between other markers.

### Survival analyses of various clinicopathological factors, biomarkers, and CD8 + TILs

As seen in Table 2, only advanced age (≥ 67 years) was significantly correlated with poorer OS in univariate COX models (p = 0.025). Multivariate analyses showed that the high Ki-67 index (HR: 7.899; p = 0.006) and advanced age (≥ 67 years) (HR: 5.899; p = 0.009) were significantly associated with poorer OS. In contrast, high CD8 + TILs (HR: 0.214; p = 0.024) and HPV-positivity (HR: 0.092; p = 0.016) were significantly associated with favorable OS. Meanwhile, poor PFS was significantly associated with PD-L1-positivity (HR: 5.311; p = 0.045), and high Ki-67 index (HR: 3.680; p = 0.042). High CD8 + TILs (HR: 0.236; p = 0.014) and HPV-positivity (HR: 0.116; p = 0.011) were also significantly associated with favorable PFS.

**Table 2:** Univariate analysis of OS and PFS

Variable	Overall survival			Progression-free survival		
	HR	95%CI	p-value	HR	95%CI	p-value
Age (< 67 vs. ≥ 67 yrs)	3.337	1.164–9.565	**0.025**	2.192	0.897–5.358	0.085
Stage (I vs. II - III)	0.829	0.430–1.596	0.574	0.855	0.476–1.539	0.620
Grade (1 vs. 2–3)	0.664	0.266–1.655	0.379	0.647	0.293–1.428	0.282
LMN (Negative vs. Positive)	1.269	0.290–5.563	0.752	2.487	0.833–7.429	0.103
HPV (Negative vs. Positive)	1.775	0.509–6.185	0.368	2.538	0.750–8.592	0.134
P53 (Wildtype vs. Mutant)	1.149	0.443–2.984	0.775	1.399	0.604–3.239	0.433
Ki-67 (Low vs. High)	1.447	0.558–3.756	0.448	1.100	0.469–2.582	0.826
TPS (< 1% vs. ≥ 1%)	1.442	0.532–3.904	0.472	1.469	0.610–3.534	0.391
CPS (< 1 vs. ≥ 1)	1.890	0.429–8.324	0.400	2.525	0.586–10.880	0.214
CD8 (Low vs. High)	0.998	0.368–2.703	0.996	0.682	0.264–1.762	0.430

### Construction of prognostic nomograms

**Fig. 1:**
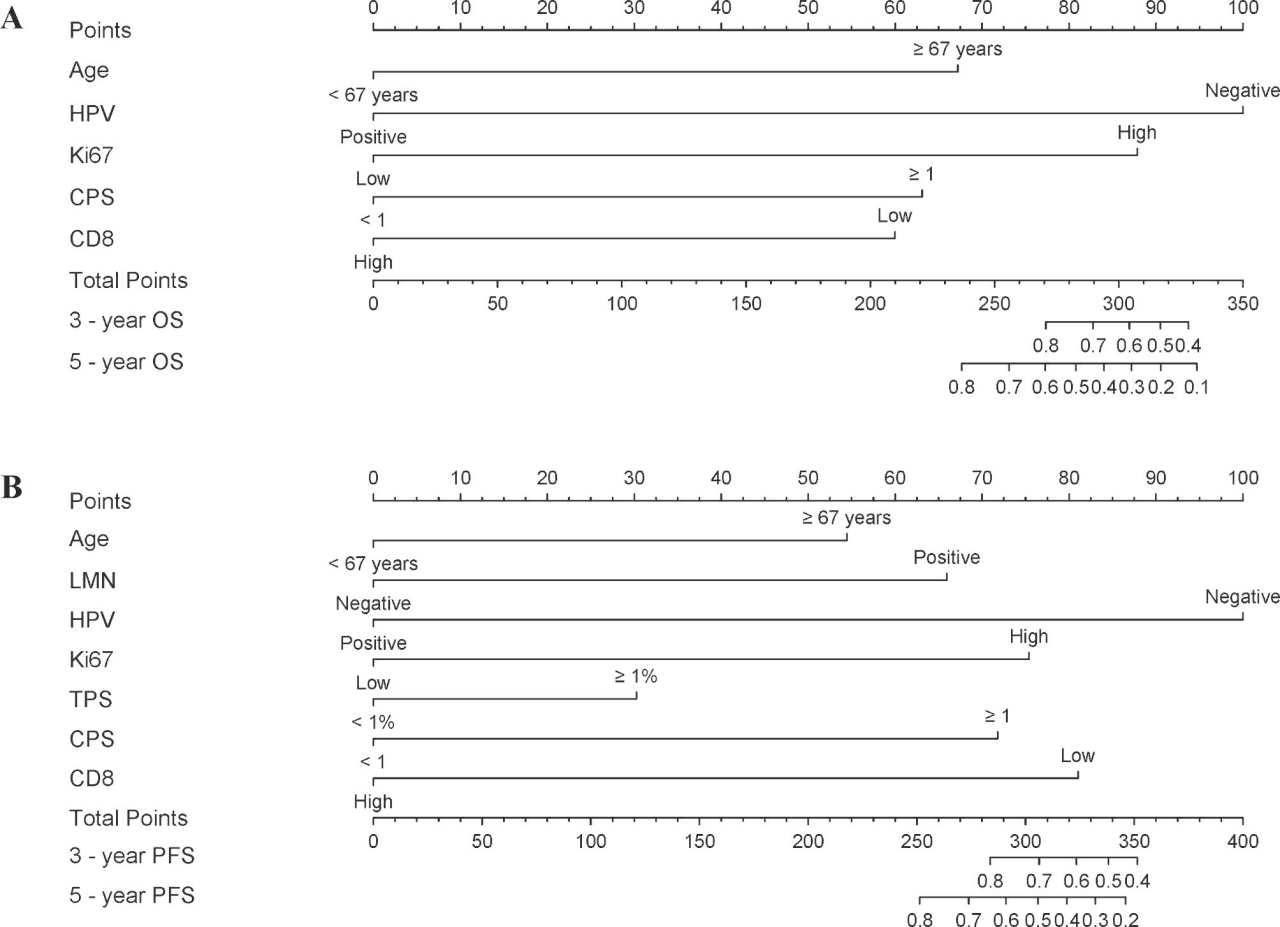
Prognostic nomograms for VSCC. (**A**) The nomogram to predict 2-year OS and 3-year OS was created based on 5 independent prognostic factors. (**B**) The nomogram to predict 2-year PFS and 3-year PFS was created based on 6 independent prognostic factors

Based on the prognostic factors from Cox models, a nomogram was constructed to predict OS and PFS (Fig. 1). To avoid the influence of the relatively small sample size, the variables with a more restricted p < 0.02 were selected to predict OS, including age (< 67 or ≥ 67 years), HPV (negative or positive), Ki-67 index (low or high), PD-L1 expression by CPS (< 1 or ≥ 1) and CD8 + TILs density (low or high). The prognostic factors selected for predicting PFS included age (< 67 or ≥ 67 years), LNM (negative or positive), HPV (negative or positive), Ki-67 index (low or high), PD-L1 expression by CPS (< 1 or ≥ 1) and CD8 TILs density (low or high). In the nomograms, a higher total score based on the sum of all factors was associated with a worse outcome. For instance, a 60-year-old patient VSCC that is HPV-negative, high Ki-67 index, PD-L1-negative, and low CD8 + TILs would have a total of 248 points (0 points for age, 100 points for HPV-negative, 88 points for high Ki-67 index, 0 points for PD-L1-negative and 60 points for low CD8 + TILs) for a predicted 2-year and 3-year OS of 91.6% and 88.7%, respectively. Similarly, a 70-year-old patient with VSCC that is HPV-negative, lymph node-positive, high Ki-67 index, PD-L1-positive, and high CD8 + TILs would have a total of 334 points (51 points for age, 100 points for HPV-negative, 53 points for lymph node-positive, 65 points for high Ki-67 index, 65 points for PD-L1 positive, and 0 points for high CD8 + TILs). For this patient, the predicted 2-year PFS was 26.7%, and the predicted 3-year PFS was 15.3%.

### Validation of prognostic nomograms

**Fig. 2:**
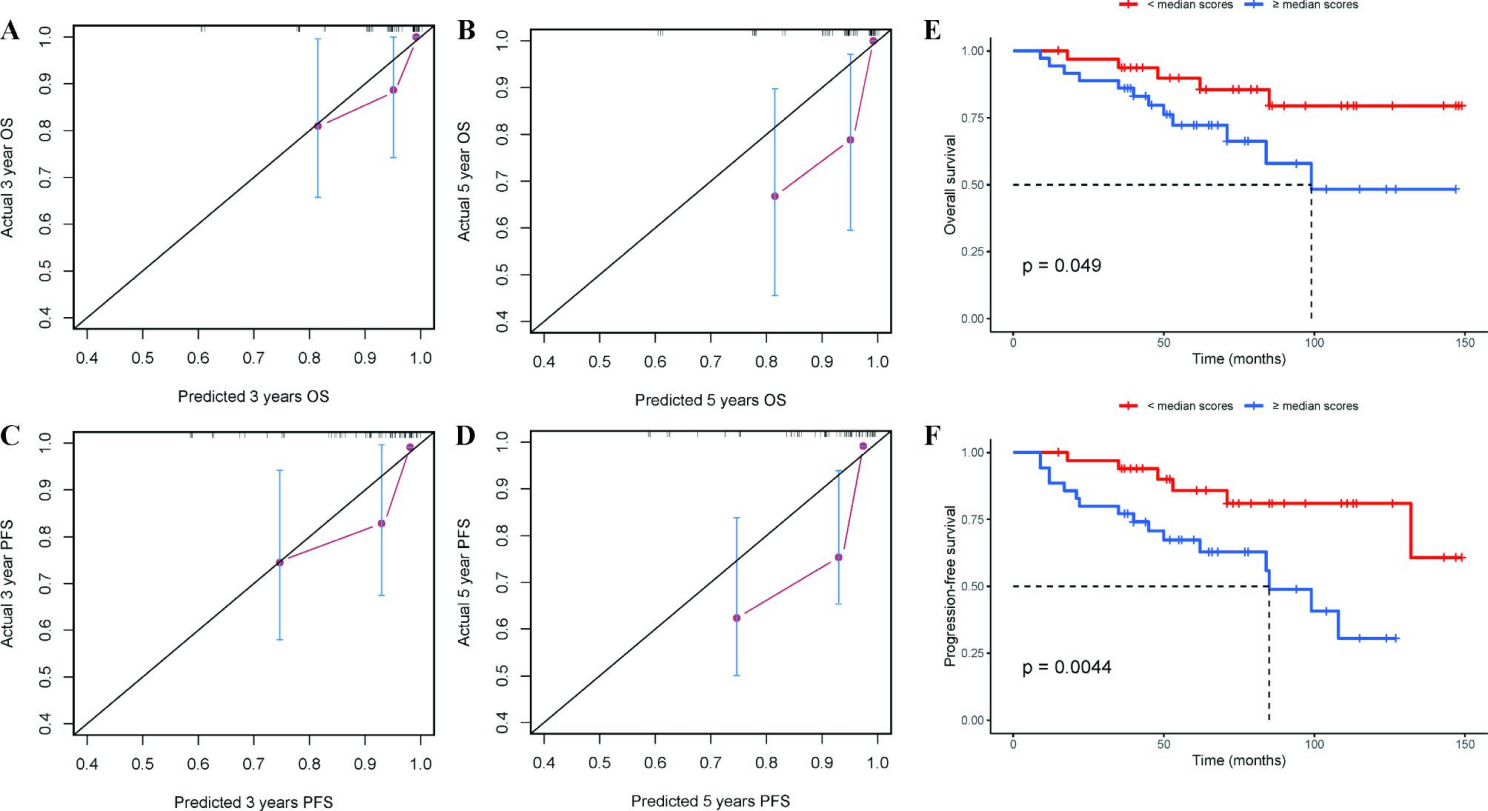
The calibration curve of the nomogram for predicting 2-year OS (**A**), 3-year OS (**B**), 2-year PFS (**C**), and 3-year PFS (**D**) in the validation cohort. The nomogram-predicted probability of OS (PFS) is plotted on the x-axis and the actual probability is on the y-axis. Kaplan-Meier curves demonstrating OS (**E**) and PFS (**F**) in VSCC patients according to mean scores of nomogram’s total points (< mean scores vs. ≥ mean scores)

VSCC patients in these nomograms had a C-index of 0.754 (95% CI [confidence interval], 0.631–0.876) and 0.754 (95% CI, 0.668–0.841) for OS and PFS, respectively. A bootstrap resampling was used to select 1,000 samples for the validation cohorts. Further testing of these nomograms’ discriminative and calibrating abilities was carried out using calibration curves for OS and PFS (Fig. 2A-D). It showed that the predicted 3-year OS and PFS models were more consistent with actual survival probability when compared with calibration plots for 5-year OS and PFS, respectively. The corrected C-index for OS and PFS of calibration curves in validation cohorts (n = 1000) were 0.699 and 0.683, respectively.

To further evaluate the performance of the nomograms, we calculated the total score for every VSCC patient by the OS and PFS model. Among patients, the median total score for OS and PFS nomogram were 230 and 216, respectively. By using median total scores as the cut-off value, patients were divided into two groups (low score or high score). Kaplan-Meier curves were presented for the survival outcome of this cohort (Fig. 2E, F). Compared with patients with low scores, patients with scores (> 230 for the OS model or > 216 for the PFS model) had a substantially worse OS (p = 0.049) or PFS (p = 0.0012).

## Discussion

Despite the downward trend of uVIN / HSIL in young women after the introduction of HPV vaccine, the downward trend did not happen in the incidence of VSCC and even significantly increased in the general population [24], which was partially driven by global aging [25]. Radical local excision is still the standard of care for VSCC, even for tumors at an early stage, which may cause high morbidity and poor quality of life. Besides traditional clinicopathological risk factors such as FIGO stage, LNM, age, etc., biomarkers also demonstrated prognostic significance in vulvar cancers [7, 17, 19, 21, 26–30]. In this study, a comprehensive approach that took consideration of all known risk factors was used in an attempt to search for better risk stratification of patients with VSCC to direct potentially less aggressive treatments in low-risk patients.

Similar to results previously reported [31], our data showed that advanced age, HPV-independent status, and positive LNM were associated with adverse survival. However, FIGO stage of the tumor showed no prognostic significance in our study. This may be due to the small size of patients with advanced stages (FIGO III and IV) in our cohort. Several studies have investigated the prognostic significance of FIGO stages of VSCC with inconsistent results. Lei L et al. established a prediction model using 2,166 VSCC patients in the SEER (Surveillance, Epidemiology, and End Results) database and also found that the FIGO staging system lost its effectiveness in evaluating prognosis[32]. In their predictive model, patients with stage III tumors had a lower prognosis score than patients with stage II tumors (higher scores indicate worse prognoses), contrary to common sense. Another study analyzed the survival predictors of 6,792 patients from the same database and also found that neither FIGO nor AJCC (American Joint Committee on Cancer) staging system had predictability of prognoses in VSCC [33]. In contrast, Jin L et al. found that stage was a strong prognostic factor for VSCC using 18,985 cases from the SEER database. It is worth noting that the staging system (SEER staging system) was used in the Jin L et al. study not based on FIGO or AJCC staging system and only divided tumors into three substages (stage I - localized, stage II - regional, or stage III - distant) [34]. Mao Y et al. observed that advanced AJCC and TNM (tumor, nodes, metastasis) stage but not SEER stage was correlated with adverse survival outcomes in their screened SEER cohorts (4681 VSCC patients) [35]. Another important prognostic clinical factor was LNM, the status of which was closely related to tumor staging, risk of recurrence, and decision on postoperative adjuvant therapy. In this study, we found that LNM was a negative predictor for PFS but not for OS in multivariate models. Many previous studies supported that LNM was strongly correlated with both OS and PFS [31, 33, 35]. Of note, a multicenter retrospective study showed that adjuvant therapy (radiotherapy or chemoradiotherapy) significantly improved the 3-year PFS in VSSC patients with LNM compared with those who did not receive postoperative adjuvant therapy [36]. However, the 3-year OS was not improved significantly [36].

In this study, we also included biomarkers: p16 and p53 associated with HPV status and classification [4, 7, 18, 21, 31, 37], Ki-67 related to tumor proliferation and differentiation [27–29, 38], and PD-L1 and CD8 + TILs linked with tumor immune microenvironment [16, 17, 19–21, 30]. The HPV status represented by p16 IHC was considered to be associated with the prognosis of VSSC, with HPV-independent tumors having worsened prognosis [31, 37, 39, 40]. The mutant p53 was commonly observed in HPV-independent dVIN or LS-associated vulvar cancers [4]. Kortekaas KE et al. further stratified p16-negative VSSC patients by using p53 IHC, and found that patients with p16-/p53mut tumor had the worst outcomes, with advanced stages, deeper invasion, and more frequent lymphovascular space invasion [7]. Ki-67 has not received as much attention as p53 in the research on vulvar cancers in recent years. It seems to the lackluster of Ki-67 was caused by its lack of correlation with tumor classification, survival, or LNM [27, 28, 41]. Stewart CJ et al. reported increased Ki-67 expression in the periphery of tumor nests and reduced at the invasive front in p16-positive VSCC. In contrast, Ki-67 expression is more diffuse in p16-negative tumors, particularly in higher-grade lesions [38]. Our findings supported p53 status as a risk stratification factor and suggested a potential role of Ki-67 in the subclassification of VSCC (Table 1). Interestingly, Ki-67 had more prognostic predictive value than p53 in both multivariate and nomogram models of our study (Table 3; Fig. 1). In addition, Kaplan-Meier curves showed no significant difference in survival between p53-negative and p53-positive in HPV- negative patients of our cohort (Figure S1). A possible cause was that VSCC patients included in this study were concentrated in stage IB and lack of more malignant events (advanced stages, LNM, etc.), which might skew the predictive sensitivity of Ki-67.

**Table 3:** Multivariate analyses of OS and PFS

Variable	Overall survival			Progression-free survival		
	HR	95%CI	p-value	HR	95%CI	p-value
Age (< 67 vs. ≥ 67 yrs)	6.335	1.670 -24.031	**0.007**	3.051	1.026–9.077	**0.045**
Stage (I vs. II - III)	0.755	0.183–3.111	0.697	0.404	0.099–1.652	0.207
Grade (1 vs. 2–3)	0.996	0.308–3.218	0.994	1.035	0.371–2.888	0.947
LMN (Negative vs. Positive)	3.045	0.440–21.072	0.259	7.334	1.319–40.768	**0.023**
HPV (Negative vs. Positive)	0.101	0.015–0.662	**0.017**	0.122	0.025–0.600	**0.010**
P53 (Wildtype vs. Mutant)	1.081	0.332–3.527	0.897	1.216	0.436–3.396	0.709
Ki-67 (Low vs. High)	9.340	2.136–40.844	**0.003**	4.348	1.237–15.283	**0.022**
TPS (< 1% vs. ≥ 1%)	2.180	0.641–7.417	0.212	2.010	0.729–5.543	0.177
CPS (< 1 vs. ≥ 1)	5.357	0.913–31.424	0.062	5.666	1.072–29.943	**0.041**
CD8 (Low vs. High)	0.169	0.042–0.682	**0.012**	0.204	0.063–0.665	**0.008**

As the most studied immune checkpoint (IC) pathway, the PD-1/PD-L1 axis function through the interaction between PD-1 present in T-cells and PD-L1 expressed in various cell types including tumor and immune cells. Success of the anti-PD-1 and anti-PD-L1 inhibitors have shown promising efficacy in many tumor types [42], including vulvar cancer [43]. We herein reported that frequent PD-L1 expression was detected in VSCC tumors and tumor-infiltrating immune cells (TPS ≥ 1% in 59.4% and CPS ≥ 1 in 82.6% of the current cohort). Our final prognostic nomograms showed that PD-L1 positivity by CPS was a strong predictor for OS and PFS. So far, PD-L1 expression in vulvar cancer has been investigated in a few studies, with conflicting results. Compared with various cutoff values of PD-L1 expression (1%, 50% for TPS and 10, 50 for CPS), Czogalla B et al. demonstrated that only PD-L1 positivity by CPS was correlated with the VSCC patients’ outcome in their study [20]. A study by Cocks M et al. in which standard TPS or CPS was not used, instead a cutoff of 1% positive cells was used, showed that PD-L1 expression was found in 43% of tumor cells, with higher proportions in intratumoral (67%) and peritumoral (81%) immune cells [30], and higher PD-L1 expression and high CD8 + TILs were associated with disease recurrence, overall mortality, and cancer mortality [30]. In contrast, the study by Sznurkowski JJ et al. suggested that immune cells with PD-L1-positivity were a favorable prognostic factor for VSCC patients [19]. Our data suggest that low CD8 + TILs as an independent prognostic factor were associated with poor OS and PFS. Unlike previously reported [19, 30, 44], no significant correlation between CD8 + TILs and PD-L1 expression was observed in this study. It is worth noting that his results from those studies were also difficult to compare as different cutoffs for the definition of PD-L1 positivity were used. Moreover, the prognostic value of CD8 + TILs was also difficult to compare because of variation in the proportion of enrolled patients with advanced diseases, which may significantly skew the result because higher CD8 + TILs were likely linked with advanced-stage cases [44].

This study has some limitations: (1) Relatively small sample size (n = 69). Future multi-center studies that include larger cohorts are needed for further validation of this study; (2) In this study, enrolled cases are mostly stage I, and stage IV case was completely missing. The composition bias of patients may skew the final statistical analysis; (3) The follow-up was limited to 1–3 years in some cases, indicating that OS and PFS were probably not reached in some cases; (4) Other clinical features such as tumor size, depth of invasion, or postoperative adjuvant therapy were not included. Future studies that address those features are needed.

## Conclusion

Inconsistent with previous studies, we found that shorter OS and PFS were associated with PD-L1-positive, higher Ki-67 expression, and low CD8 + TILs. LNM was only linked with poor PFS in VSCC patients. In addition, our study explored the main risk factors of VSCC from clinicopathological parameters and immunohistochemical panels by prognostic nomograms. Although our conclusions need to be confirmed by future studies with larger samples sizes, this data adds to the continued efforts of better risk stratification of VSCC patients, in hope of potentially less aggressive treatments in low-risk patients to decrease morbidity and improve quality of life.

## Electronic supplementary material

Below is the link to the electronic supplementary material.

Supplementary Material 1

Supplementary Material 2

## Data Availability

Data supporting the findings of this study are available upon request from the corresponding author.

## Abbreviations

SCC: squamous cell carcinoma
VSCC: vulvar squamous cell carcinoma
CSCC: cervical squamous cell carcinoma
OS: overall survival
PFS: progression-free survival
HR: hazard ratio
CI: confidence interval
TILs: tumor-infiltrating lymphocytes
CPS: combined positive score
uVIN: usual vulvar intraepithelial neoplasia
HSIL: high-grade squamous intraepithelial lesion
dVIN: differentiated vulvar intraepithelial neoplasia
hrHPV: high-risk human papillomavirus
LS: lichen sclerosus
FIGO: International Federation of Gynecology and Obstetrics
LNM: lymph node metastasis
IHC: immunohistochemistry
SEER: Surveillance, Epidemiology, and End Results
AJCC: American Joint Committee on Cancer
IC: immune checkpoint
