# Supplementary material for: An integrated model for prognosis in vulvar squamous cell carcinoma
Source: BMC Cancer. 2023 Jun 12;23:534. doi: 10.1186/s12885-023-11039-2 (PMC10259032; doi:10.1186/s12885-023-11039-2)
Supplement: Supplementary file 1 — Supplementary Material 1 [file 12885_2023_11039_MOESM1_ESM.docx]

| **Table S1. Association of clinicopathological features** | | | | | |
| --- | --- | --- | --- | --- | --- |
| p r | Age | Stage | Grade | LMN | HPV-status |
| Age |  | -0.008 | -0.071 | -0.107 | -0.207 |
| Stage | 0.948 |  | -0.041 | **0.440** | -0.170 |
| Grade | 0.560 | 0.740 |  | -0.009 | 0.121 |
| LMN | 0.380 | **< 0.001** | 0.939 |  | 0.051 |
| HPV-status | 0.088 | 0.163 | 0.324 | 0.679 |  |

The blue part showed the p values, and the green part showed the correlation coefficients (using Spearman's test)
