# Supplementary material for: An integrated model for prognosis in vulvar squamous cell carcinoma
Source: BMC Cancer. 2023 Jun 12;23:534. doi: 10.1186/s12885-023-11039-2 (PMC10259032; doi:10.1186/s12885-023-11039-2)
Supplement: Supplementary file 2 — Supplementary Material 2 [file 12885_2023_11039_MOESM2_ESM.docx]

**
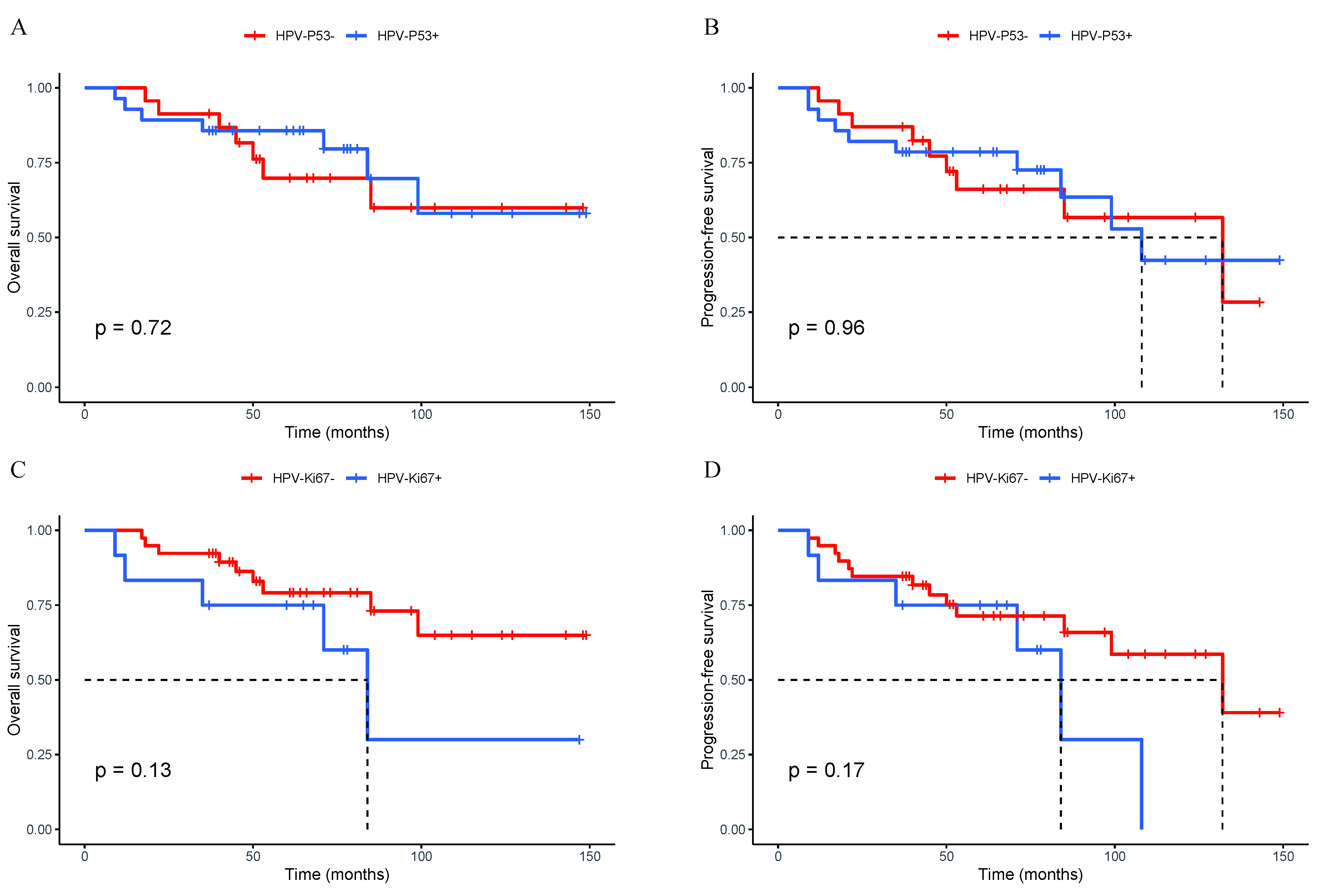
**

**Figure S1.** Kaplan–Meier survival analyses of markers in HPV-negative patients. Comparison of OS (A) and PFS (B) by p53 mutant status (p53Wt [wildtype] vs. p53Mut [mutant]).
